# Expression of neutral endopeptidase activity during clinical and experimental acute lung injury

**DOI:** 10.1186/1465-9921-11-164

**Published:** 2010-11-29

**Authors:** Soshi Hashimoto, Fumimasa Amaya, Kentaro Oh-hashi, Kazutoshi Kiuchi, Satoru Hashimoto

**Affiliations:** 1 Department of Anesthesiology and Intensive Care, Kyoto Prefectural University of Medicine, Kyoto, Japan; 2 Department of Biomolecular Science, Faculty of Engineering, Gifu University, Gifu, Japan

## Abstract

**Background:**

Neutral endopeptidase (NEP), an enzyme that cleaves inflammatory bioactive peptides, may play a protective role in the pathogenesis of acute lung injury (ALI) and acute respiratory distress syndrome (ARDS). However, its low extracellular activity hinders the precise measurement of changes that take place during ALI/ARDS. The main objective of this study was to clarify the regulation of NEP activity and its expression during ALI/ARDS.

**Methods:**

In a *clinical study*, we measured plasma NEP activity in patients who developed postoperative ALI/ARDS, using a HPLC fluorometric system. In an *experimental study*, we induced ALI by intratracheal instillation of hydrochloric acid (HCl) or lipopolysaccharide (LPS) in mice, and similarly measured NEP activity in plasma, lung tissue, and broncho-alveolar lavage fluid (BALF). We also studied the distribution and measured the amounts of NEP protein, using immuno-histochemical and immunoblot analyses, and measured the levels of NEP mRNA, using real-time reverse transcription-polymerase chain reaction, in the lungs of mice with ALI.

**Results:**

The plasma NEP activity was significantly lower in patients presenting with ALI/ARDS than in controls. Similarly, the NEP activity in plasma and lung tissue was markedly lower, and lung injuries more severe in LPS- than in HCl-treated mice. In contrast, the activity of NEP in the BALF of LPS-treated mice was increased. The intratracheal instillation of LPS decreased the gene expression of NEP in the lung. Immuno-histochemical and Western immunoblot studies in mice confirmed a) the presence of NEP in the alveolar wall, a critical target in ALI/ARDS, and b) a decrease in its expression in HCl- and LPS-induced ALI.

**Conclusion:**

In this experimental and clinical study of ALI/ARDS, the activity of NEP was significantly decreased in plasma and increased in the alveolar air space.

## Background

Acute respiratory distress syndrome (ARDS), the most severe form of acute lung injury (ALI), is associated with an up to 35% mortality rate, despite intensive care [1,2]. Severe sepsis, pneumonia, acid aspiration, and surgical stress are the major causes of ALI/ARDS, which disrupt the alveolar-capillary membrane barrier and induce pulmonary edema associated with proteinaceous alveolar exudates and deterioration of gas exchange [3]. While several biological mediators are known to make important contributions to its development, the interactions among cellular and soluble mediators responsible for ALI/ARDS are complex and incompletely understood.

Neutral endopeptidase (NEP; EC 3.4.15.1), also known as enkephalinase, neprilysin, common acute lymphoblastic leukemia antigen (CALLA) or CD10, is a membrane-bound zinc-metallopeptidase enzyme that regulates the physiological action of various peptides, including natriuretic and bombesin-like peptides, endothelin-1, and substance P [4-6]. The involvement of NEP in the limitation of pro-inflammation has been studied, using selective inhibitors and gene deletion in mice. NEP-deficient mice develop exaggerated LPS-induced endotoxic shock [7] and acute pancreatitis-associated lung injury [8]. The pharmacological inhibition of NEP exacerbates ALI in mice presenting with fire-smoke and fire-burn injuries [9]. Recent studies have also found a decreased NEP activity participating in vascular permeability and edema formation in the lung [9,10]. Other metallopeptidases present in the lung, such as angiotensin- and endothelin-converting enzymes, share some substrates with NEP [6], and may be inversely implicated in the development of ALI/ARDS [11-13].

While evidence is accumulating of an important protective role played by NEP, the measurements of its activity in ALI/ARDS have been ambiguous, probably because of the low sensitivity of conventional photometric measurements. A deeper understanding of the regulation of NEP activity in ALI/ARDS would yield valuable mechanistic information. Thus, the objectives of this study were to 1) measure the activity of NEP in clinical and experimental lung injury, and 2) clarify the systemic and intra-alveolar regulation of NEP activity in ALI/ARDS, using a newly developed high-sensitivity measurement system.

## Methods

### Patient population

The clinical study was conducted in the general intensive care unit of the Kyoto Prefectural University of Medicine Hospital, using protocols approved by the Ethics Committee on Human Research. We studied 8 randomly selected patients, who underwent total thoracic esophagectomy and reconstruction with a retrosternal stomach tube for esophageal cancer. Diagnoses of ALI or ARDS were made on the basis of the definitions formulated by the American European Consensus Conference [14]. A lung injury score was also calculated for each patient, according to published algorithms [15]. Blood samples were collected in sterile heparinized tubes and centrifuged at 500 g for 10 min. Plasma samples were stored at -80°C until analysis.

### Animal preparations

Male specific pathogen-free ICR mice, 6-8 weeks of age, were obtained from Japan SLC Inc., Shizuoka, Japan. The mice were housed under standard laboratory conditions and allowed free access to food and water. All procedures were carried out in compliance with the guidelines approved by the Institutional Animal Care and Use Committees of Kyoto Prefectural University of Medicine.

### Induction of lung injury

The mice were anesthetized with sevoflurane, and received a single intratracheal instillation of 2 ml/kg of normal saline, or 0.1 N hydrochloric acid (HCl), or 10 mg/kg lipopolysaccharide (LPS) in 2 ml/kg of normal saline, using a previously described procedure [16]. The HCl-treated animals were sacrificed at 4 h, and the LPS-treated or control mice were sacrificed at 24 h after instillation, all under deep anesthesia with sevoflurane.

### Lung histology and immunohistochemistry

Paraformaldehyde-fixed, paraffin-embedded sections of mouse lung tissue were prepared and stained with hematoxylin and eosin. The microscopic slides were examined by an investigator unaware of their origin, who assigned a lung injury score in 4 categories, including the thickness of the alveolar septa, presence of alveolar hemorrhage, formation of hyaline membrane, and presence of intra-alveolar infiltrates. The severity of injury was judged according to a previously published scoring system [17], between 0 = no or minimal injury, 1 = mild injury, 2 = moderate injury, 3 = severe injury, and 4 = maximum injury. For the immuno-histochemistry, the antigen was retrieved with citrate buffer and the tissue blocked with 1% H_2_O_2_, avidin, biotin, and mouse-on-mouse blocking reagent (Vector Lab, Burlingame, CA), as appropriate. The sections were incubated with mouse anti-NEP (Mitsubishi Chemical Co., Tokyo, Japan) followed by a biotinylated secondary antibody. The signals were detected by the ABC method (Vector), followed by diaminobenzidine (Dako Japan Ltd., Kyoto, Japan). The slides were counterstained with hematoxylin.

### Lung water content

The lung water produced by the injury was measured, using the lung wet/dry (W/D) weight ratio. The right lobe was dissected, weighed immediately after removal, and dried for 5 days in an oven at 60°C. The lung W/D weight ratio was calculated as the ratio of wet to dry weight.

### Broncho-alveolar lavage analyses

The broncho-alveolar lavage consisted of 2 washes with 1.0 ml of phosphate buffered saline. The broncho-alveolar lavage fluid (BALF) was centrifuged at 400 g for 10 min, and the cell pellet was resuspended in 1.0 ml of phosphate buffered saline. The total number of cells in the lavage fluid was counted with a hemocytometer. Cell differentials were counted on cytospin preparations stained with Diff-Quik (Sysmex, Kobe, Japan). At least 200 cells per sample were counted under light microscopy. The cytospin preparations from the lavage were also fixed with 4% paraformaldehyde for 10 min, followed by immuno-cytochemical staining for NEP as described earlier. The cell-free supernatant of BALF was immediately stored at -80°C for total protein and NEP activity assays.

### Measurements of NEP activity

The NEP activity was measured as described previously [18]. An aliquot of each humoral sample was incubated with 200 μM of Dansyl-D-Ala-Gly (DAG) *p*-nitro-Phe-Gly (NPG) in presence or absence of 10 μM of thiorphan, at 37° C for 60 min. The enzymatic reaction was terminated by heating at 90° C for 3 min. After brief centrifugation, an aliquot of the supernatant was analyzed by HPLC-fluorometry. The fluorescence intensity of DAG separated from the fluorescence of the substrate was measured, using a RF-10AXL, fluorescence detector for HPLC (Shimadzu). A reverse, 4.6 mm ϕ × 250 mm Inertsil-3 C18 gel column (GL Science), with a guard column was used for the assay. The injected sample was eluted with 40% acetonitrile containing 0.05% trifluoroacetic acid, at a flow rate of 0.5 ml/min at 40° C. The amount of product was estimated by measuring its fluorescence intensity at 562 nm (Em) with excitation at 342 nm (Ex). For measurements of NEP activity in mouse lungs, each specimen was homogenized with 5 volumes of 50 mM homogenate HEPES buffer at pH 7.4, containing 1 mM ethylene glycol tetraacetic acid, 50 μM phenylmethylsulfonyl fluoride, 1 μg/ml pepstatin and 1 μg/ml leupeptin, and centrifuged at 1,000 g for 5 min. An aliquot of supernatant was incubated with this homogenate buffer containing 200 μM of DAGNPG, at 37°C for 30 min, and the fluorescence intensity of DAG was measured as described earlier. The specific NEP activity was estimated after the measurement of protein concentration in each sample, according to the method of Bradford.

### Immunoblot analysis

We added 25 μg of protein to Laemmli sample buffer, boiled for 5 min. The proteins extracted from each lung tissue were electrophoresed on 12.5% sodium dodecyl sulfate-polyacrylamide gels followed by transfer to a polyvinylidene fluoride membrane (Millipore, Bedford, MA). A horseradish peroxidase-conjugated secondary antibody and ECL™ Advance Detection Kit (GE Healthcare Bio-Sciences, Piscataway, NJ) were used for immunodetection. β-actin (Santa Cruz Biotechnology, Santa Cruz, CA) was used as loading control, and the intensity of the NEP signal was semi-quantified from scanned immunoblots, using Adobe^® ^Photoshop^® ^software (Adobe Systems Inc., San Jose, CA).

### Real-time polymerase chain reaction

Total RNA was extracted from lung tissue using a TRIzol^® ^reagent (Invitrogen Life Technologies, Carlsbad, CA). Total RNA was reverse-transcribed to cDNA with SuperScript^® ^III (Invitrogen) first-strand synthesis for real-time reverse transcription (RT) -polymerase chain reaction (PCR). The primer sequences for NEP and 18 S rRNA are available upon request (Takara Bio Inc, Tokyo, Japan). RT quantitative PCR was performed using the 7300 Real-Time PCR System (Applied Biosystems, Foster City, CA) and Platinum^® ^SYBR^® ^Green qPCR SuperMix-UDG (Invitrogen). The relative mRNA expression levels were calculated by the relative standard curve method and normalized to 18 S rRNA.

### Statistical analysis

The results are expressed as means ± SEM. Between-groups comparisons were made, using analysis of variance or Student's *t*-test, as appropriate. A p value < 0.05 was considered significant.

## Results

### NEP activity in plasma of patients with ALI/ARDS

**Figure 1:**
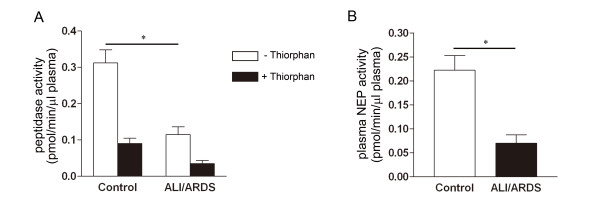
**NEP activity in human plasma**. Plasma NEP activity in Controls versus patients who developed ALI/ARDS. **A**. Peptidase activity in absence (open column) or presence (filled column) of thiorphan. **B**. NEP activity calculated by subtraction of the thiorphan-insensitive activity from the total activity. n = 4 in each group, *p < 0.05 vs. Control.

Selected characteristics of the 8 patients are shown in Table 1. Peptidase activity was estimated by measuring the fluorescence intensity of the cleavage product separated from the fluorescence substrate by HPLC. Figure 1 shows the NEP activity levels in the plasma from patients without ALI (control group) and from patients with ALI/ARDS. The mean ± SEM peptidase activity measured with DAGNPG in 4 control patients was 0.31 ± 0.04 pmol/min/μl in absence, and 0.09 ± 0.01 pmol/min/μl in presence of thiorphan. In the 4 patients who developed ALI/ARDS, the corresponding values were 0.12 ± 0.02 pmol/min/μl in absence, versus 0.04 ± 0.01 pmol/min/μl in presence of thiorphan. The difference between the 2 groups in total activity in absence of thiorphan was significant (p = 0.0031), whereas in presence of thiorphan the total activity was similar in both groups (Figure 1A). Cleavage of the fluorescent substrate was suppressed in presence of thiorphan, a relatively specific inhibitor of NEP, which inhibits a few enzymes other than NEP. The activity of thiorphan-insensitive enzyme measured in presence of thiorphan was subtracted from the total activity to calculate the activity of NEP. The NEP activity measured in the control and the ALI/ARDS groups was 0.22 ± 0.03 and 0.07 ± 0.02 pmol/min/μl, respectively (Figure 1B). The difference between the 2 groups in plasma NEP activity was also significant (p = 0.0049).

**Table 1:** Selected characteristics of the study patients

Group	Sex	Age (y)	PaO_2_/FiO_2_	Lung injury score	Outcome
**Control**	Male	63	386	0.3	Alive
**Control**	Female	56	325	0.3	Alive
**Control**	Female	74	440	0.3	Alive
**Control**	Male	70	386	0	Alive
**ALI/ARDS**	Male	73	148	2.3	Alive
**ALI/ARDS**	Male	77	103	2.0	Deceased
**ALI/ARDS**	Male	71	133	2.0	Deceased
**ALI/ARDS**	Male	64	118	2.3	Alive

### Induction of lung injury in mice

**Figure 2:**
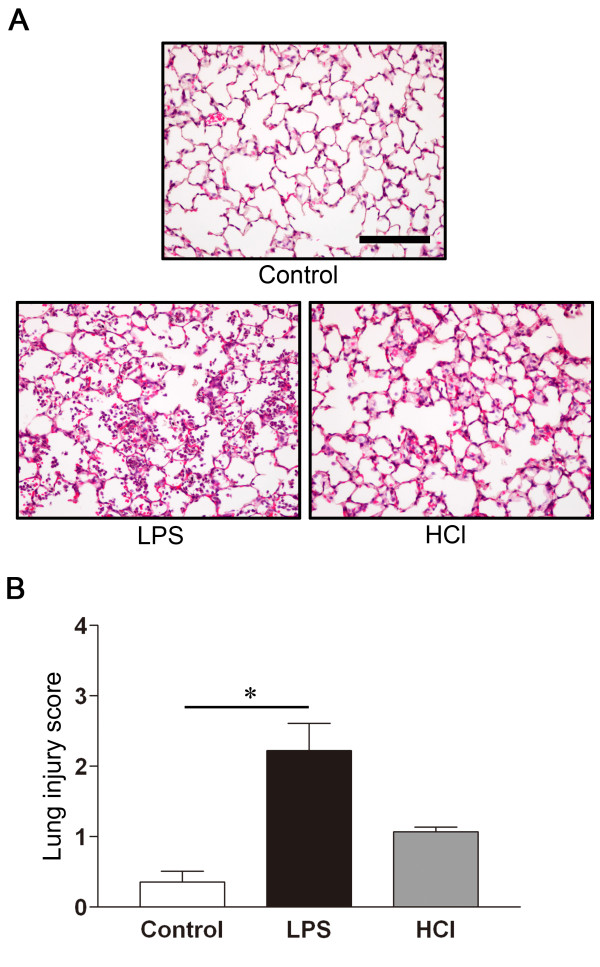
**Histologic evaluation of lung injury in mice**. **A**. Representative lung sections from mice after single intratracheal instillation of normal saline, or 2 ml/kg HCl, or 10 mg/kg LPS. The LPS-treated or control mice were sacrificed at 24 h, and the HCl-treated animals were sacrificed at 4 h after instillation, and left lung lobes were taken from mice. The lung sections were stained with hematoxylin and eosin and are shown at X 200 original magnification (bar = 50 μm). **B**. Degree of lung injury ascertained, using the lung injury scoring system. n = 5 in each group, *p < 0.05 vs. Control.

**Figure 3:**
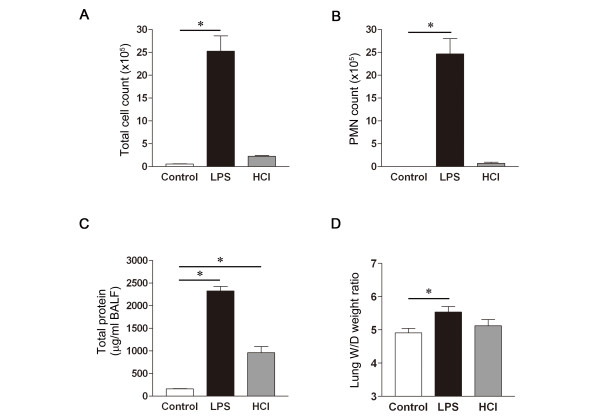
**BALF analysis and W/D weight ratio after induction of lung injury in mice**. **A**. Total cell count in BALF counted by hemocytometry. **B**. PMN count. Diff-Quik stain under light microscopy. **C**. Total protein concentration in BALF as an indicator of alveolar barrier dysfunction. **D**. Pulmonary edema caused by intratracheal instillation of LPS or HCl formation measured, using the lung wet/dry (W/D) weight ratio. n = 4 to 6 in each group, *p < 0.05 vs. Control.

To ascertain the severity of lung injury, we examined the lung tissue histology, the cell count and total protein concentration in BALF, and the W/D weight ratio. While only mild alveolar thickening and cellular infiltration were observed in the HCl-treated mice, more prominent alveolar distortion and cellular infiltration were present in the LPS-treated mice. Minimal histological changes were observed in the control group (Figure 2A). The histopathologic severity of lung injury was also scored semi-quantitatively. The lung injury score was significantly higher in the LPS- than in the HCl-treated and the control mice (Figure 2B). Furthermore, the total cell (Figure 3A) and polymorphonuclear (PMN) leukocytes (Figure 3B) counts in BALF were significantly higher in the LPS- than in the HCl-treated and the control mice, and were similar in the HCl-treated and the control mice. The total protein concentration in the BALF from the LPS- and HCl-treated mice was significantly higher than in the BALF from the control mice (Figure 3C). The W/D weight ratio, as an indicator of lung edema, was also significantly higher in the LPS-treated than in the control group. Although the W/D weight ratio was higher in the HCl-treated than in the control mice, the difference was not statistically significant (Figure 3D).

### Immuno-histochemical and immuno-cytochemical staining for NEP

**Figure 4:**
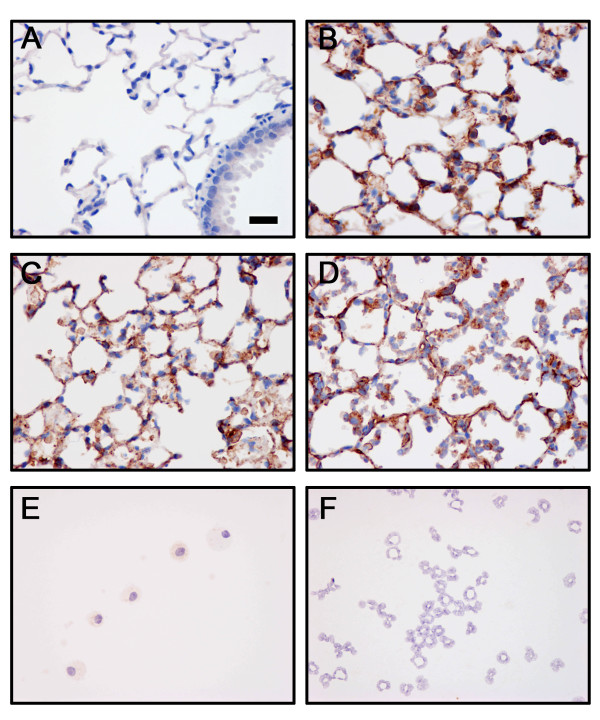
**Immuno-histochemical and immuno-cytochemical studies with normal IgG as a negative control (A), and with a monoclonal antibody against NEP (B-F)**. **A**. No staining was observed in normal lung, using non-immune IgG as negative control. **B**. Prominent staining for NEP was observed in the alveolar wall, particularly in normal type II alveolar epithelium. A decreased immunoreactivity for NEP was apparent in the lungs, 4 h after the instillation of HCl **(C)**, and 24 h after the instillation of LPS **(D)**. Immunocytochemistry of BALF cytospin preparations showed a low immunoreactivity for NEP in resident alveolar macrophages from control mice (**E)**, and infiltrating PMN leukocytes from LPS-treated mice (**F)**. Representative photomicrographs of lung sections and cytospin preparations from groups of 4 or 5 mice at X 400 original magnification (bar = 20 μm).

The distribution of NEP in the lungs was examined by immuno-histochemistry, and the pattern of NEP expression in the lung of mice is shown in Figure 4. In normal lungs, NEP staining was localized prominently in the alveolar wall, particularly in type II and probably in type I epithelial cells (Figure 4B), as well as in the epithelial and smooth muscle cells of the airways, as identified previously [19]. No staining was observed when non-immune IgG was used as a negative control (Figure 4A). After the induction of ALI, the intensity of staining for NEP in the alveolar wall appeared lower with the destruction of the walls and sloughing of the epithelial cells. Although the intratracheal instillation of both LPS and HCl recruited PMN into the lung, the immuno-histochemical expression of NEP in the injured lungs showed less intense staining of the PMN than of the alveolar walls (Figure 4C &4D). Immuno-cytochemistry of the BALF cytospin preparations also showed that the expression of NEP in infiltrating inflammatory cells (Figure 4F) and resident alveolar macrophages (Figure 4E) was low.

### Decreased plasma NEP activity in mice with acute lung injury

**Figure 5:**
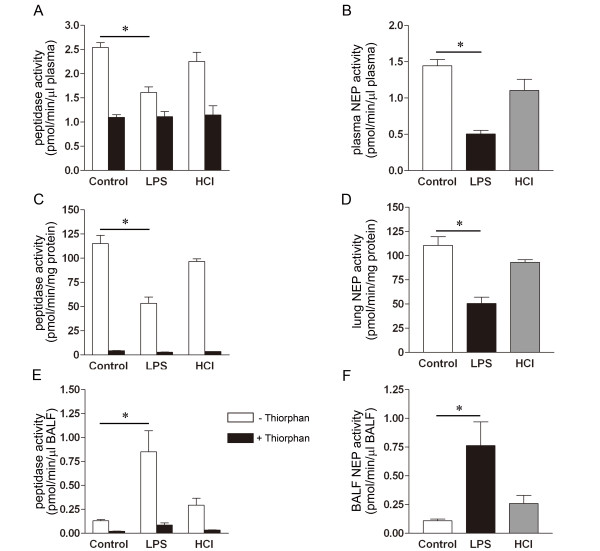
**NEP activity in mice measured in plasma (A & B), lung tissue (C & D), and BALF (E & F), after the induction of lung injury in mice**. Peptidase activity of plasma (A), lung tissue (C), and BALF (E) in absence (open column) versus presence (solid column) of thiorphan. NEP activity of plasma (B), lung tissue (D), and BALF (F) calculated by subtraction of thiorphan-insensitive from total peptidase activity. n = 3 to 5 in each group, *p < 0.05 vs. Control.

The mean ± SEM peptidase activity measured in 5 control mice was 2.54 ± 0.10 pmol/min/μl in absence, and 1.10 ± 0.05 pmol/min/μl in presence of thiorphan. The corresponding measurements in 5 LPS-treated mice were 1.61 ± 0.12 and 1.11 ± 0.11 pmol/min/μl, respectively, versus 2.25 ± 0.19 and 1.15 ± 0.19 pmol/min/μl, respectively, in 3 HCl-treated mice (Figure 5A). As was observed in patients, the plasma NEP activity was lower in 5 LPS-treated (0.50 ± 0.05 pmol/min/μl) than in 5 control (1.45 ± 0.09 pmol/min/μl) mice. The difference in plasma NEP activity between 3 HCl-treated (1.10 ± 0.15 pmol/min/μl) and the 5 control (1.45 ± 0.09 pmol/min/μl) mice was not statistically significant (Figure 5B).

### Decreased lung NEP activity in mice with acute lung injury

The mean ± SEM peptidase activity measured in 5 control mice was 115 ± 8.6 pmol/min/mg in absence, and 4.32 ± 0.36 pmol/min/mg in presence of thiorphan. The corresponding measurements in 4 LPS-treated mice were 54 ± 6.4 and 2.83 ± 0.21 pmol/min/mg, respectively, versus 96 ± 2.9 and 3.53 ± 0.03 pmol/min/mg, respectively, in 3 HCl-treated mice (Figure 5C). The NEP activity level in lung tissue, like in plasma, was lower in 4 mice with ALI induced by LPS (51 ± 6.5 pmol/min/mg) than in 5 control mice (111 ± 8.8 pmol/min/mg). The lung NEP activity levels in 3 HCl-treated (93 ± 2.8 pmol/min/mg) and 5 control mice (111 ± 8.8 pmol/min/mg) were similar (Figure 5D).

### Elevated NEP activity in the BALF of mice with acute lung injury

NEP activity was detectable in the BALF of all animals. However, in contrast to the NEP activity in plasma, that in BALF was nearly 7-fold higher in 5 LPS-treated (0.76 ± 0.21 pmol/min/μl) than in 5 control (0.11 ± 0.01 pmol/min/μl) mice. The NEP activity in 5 HCl-treated mice was higher (0.26 ± 0.07 pmol/min/μl) than in the 5 control (0.11 ± 0.01 pmol/min/μl) mice, though the difference was not statistically significant (Figure 5E &5F). In all experimental groups, the thiorphan-insensitive activity levels in plasma, lung homogenate, and BALF were similarly low.

### Decreased NEP protein in the lung tissue of mice with acute lung injury

**Figure 6:**
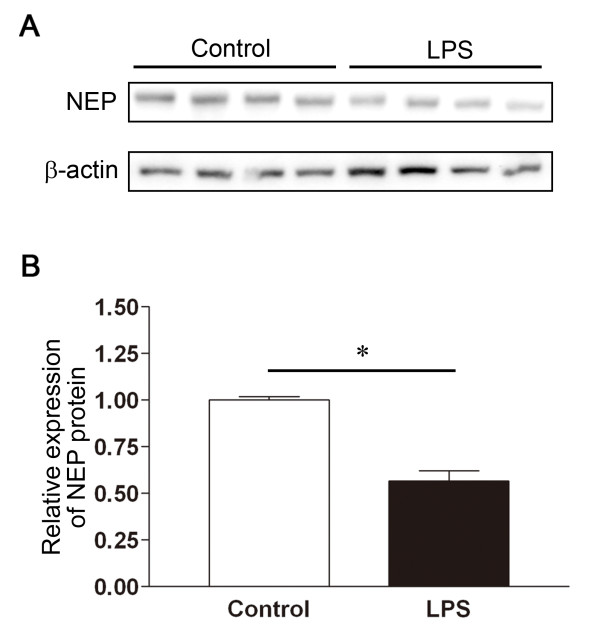
**Relative expression of NEP protein in lung tissue**. **A**. Representative whole lung tissue immunoblot for NEP and β-actin, in a control preparation versus 24 h after the instillation of LPS. **B**. Densitometric measurements of NEP expression in 5 control versus 5 LPS-treated mice. *p < 0.05.

To determine whether the decrease in lung NEP activity associated with ALI was due to a decrease in the expression of NEP protein in the lung, we measured the NEP protein content in lung homogenates using Western immunoblotting, and found that the expression of NEP protein in the lung in LPS-induced ALI was < 60% of the expression measured under control conditions (Figure 6).

### Inhibition of NEP gene expression in the lung tissue of mice with acute lung injury

**Figure 7:**
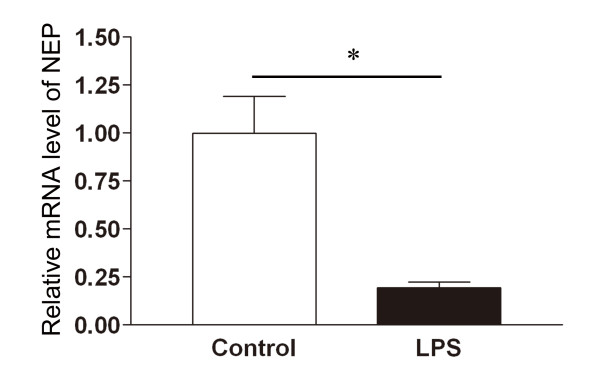
**Relative mRNA expression of NEP in lung tissue**. Relative gene expression patterns of NEP in whole lung tissue, 24 h after the instillation of LPS versus saline (Control), analyzed by quantitative RT-PCR. The results are expressed as relative amounts normalized for 18 S rRNA. n = 4 or 5 mice in each group. *p < 0.05.

To study the transcriptional regulation of the NEP gene, we also measured the NEP mRNA levels in lung tissue of mice with ALI, using semi-quantitative RT-PCR. The NEP mRNA levels were significantly lower in the lungs of 5 LPS-treated mice than in the lungs of 4 mice treated with saline alone (Figure 7).

## Discussion

In this clinical and experimental study of ALI, the plasma NEP activity was prominently decreased in patients as well as in mice. In mice, the downregulation of NEP activity in plasma and lung was associated with a decrease in the amounts of protein and gene expression of NEP in lung tissue. We also found an increase in NEP activity in the intra-alveolar space of mice with ALI, in contrast to its activity in plasma. Our immuno-histochemical observations localized the NEP protein in the alveolar wall, where its expression was decreased by ALI, suggesting that the injured alveolar wall is a major source of intra-alveolar NEP activity.

In contrast to previous observations [20], we found that the activity of NEP in plasma was lower in presence of ALI. On the other hand, a decrease in NEP activity in plasma has been reported during septic shock, a major cause of ALI/ARDS [21]. To further study this apparent conflict, we used a HPLC-fluorometric method, which we found, in earlier studies, more sensitive to measure the extracellular activity of NEP on the cell surface [22,23]. The measurements made with this sensitive method indicated that the activity of NEP in plasma was decreased during ALI. The significant decrease in the NEP enzymatic activity in the lung of mice with ALI was associated with a lower level of its gene expression in the lung, suggesting that the downregulation of NEP activity was due to its decreased expression at the transcriptional level responsible for ALI/ARDS. This downregulation of NEP activity may contribute to the inactivation of the tachykinins degradation pathway and to uncontrolled inflammation in ALI/ARDS, and in other neurogenic respiratory diseases [9,24-26].

Our measurements of NEP activity were based on the cleavage products of the fluorescence substrate suppressed by thiorphan, a specific inhibitor widely used to measure the activity of NEP in various samples [27]. Among the metalloproteases, angiotensin-converting enzyme [28], endothelin-converting enzyme [29] and insulin-degrading enzymes [30] are only slightly inhibited by thiorphan. On the other hand, thiorphan inhibits the activities of NEP2 [22], KELL [31], DINE [32] and PEX [33]. We have also reported that NEP2 is sensitive to thiorphan, though it does not cleave DAGNPG [22], a substrate used in the present study. The substrate specificities of KELL, DINE and PEX remain to be determined.

We used 2 murine models of ALI to measure the activity of NEP. The intratracheal instillation of LPS and HCl aspiration are forms of lung injury which are both neutrophil-dependent, and are characterized by inflammation in the alveolar space and injury to the alveolar barrier, associated with an increase in endothelial and epithelial permeability [34]. Compared with the instillation of HCl, LPS caused more prominent alveolar destruction, greater alveolar recruitment of PMN, and more severe alveolar exudates. These differences in severity of lung injury may be due to the measurements made 4 h after the instillation of HCl versus 24 h after that of LPS. The systemic downregulation and intra-alveolar upregulation of NEP activity were also more marked in the LPS group; therefore, the regulation of its activity may be modulated in response to the severity of lung injury. It is also noteworthy that the urinary excretion of NEP been suggested as a new biomarker of acute renal injury occurring after cardiac surgery [35].

Our immuno-histochemical studies showed that NEP was expressed in the alveolar wall, most prominently in type II alveolar epithelium, and that this expression was decreased by the epithelial sloughing associated with ALI. Decreased amounts of NEP protein in the lung were also confirmed by Western immunoblot analysis. The increased activity of NEP in BALF might, therefore, reflect alveolar injury with secondary shedding from the cell surface. In support of this hypothesis, it has been suggested that the injury due the inhalation of fire smoke causes the shedding of NEP from the airways' epithelium [19]. Likewise, studies of diabetic retinopathy and idiopathic arthritis found that the tissue injury due to the inflammatory response can increase the extracellular NEP activity in the interstitial space [23,36]. A recent study has also found an elevated NEP activity in the cerebro-spinal fluid of patients suffering from Alzheimer's disease, attributed to synaptic disruption [37]. An alternate explanation for the increase in NEP activity in BALF may be the extravascular leakage of its soluble isoform in response to the alveolar-capillary barrier injury.

We did not exclude intra-alveolar inflammatory cells, including PMN, as the source of increased NEP activity in BALF. Previous studies have observed the expression of NEP on the surface of PMN [38,39]. In addition, the NEP activity was higher in the LPS-treated mice, in which the recruitment of PMN into the air-space was increased. However, our immuno-histochemical and immuno-cytochemical analyses showed prominent NEP staining neither on inflammatory cells recruited into the air-space, nor on resident alveolar macrophages. A previous study found that the expression of NEP on circulating PMN was reduced in humans challenged with LPS [40]. Thus, the NEP expression was most likely down-regulated on the PMN that were recruited and activated during ALI/ARDS.

### Study limitations

The major limitations of our clinical study are its small sample size and retrospective design, due to our intent to study a homogeneous population of postoperative patients with ALI/ARDS. Therefore, we supplemented our investigations of the regulatory mechanisms of NEP activity by adding the mice experiments. Despite the limited number of patients, this study offers new insights into the pathogenesis of ALI/ARDS. A larger, randomized trial will be needed to confirm the existence of a correlation between NEP activity and severity of ALI/ARDS.

## Conclusions

We found, in ALI/ARDS, a significant decrease in the plasma activity of NEP, while its intra-alveolar activity was significantly increased. The identification of the enzymatic regulation associated with NEP would broaden our understanding of the role it plays in ALI/ARDS.

## Abbreviations

NEP: neutral endopeptidase; ALI: acute lung injury; ARDS: acute respiratory distress syndrome; HCl: hydrochloric acid; LPS: lipopolysaccharide; CALLA: common acute lymphoblastic leukemia antigen; BALF: broncho-alveolar lavage fluid; DAGNPG: Dansyl-D-Ala-Gly-*p*-nitro-Phe-Gly; DAG: Dansyl-D-Ala-Gly; HPLC: high performance liquid chromatography

## Competing interests

The authors declare that they have no competing interests.

## Authors' contributions

SoH conducted the experimental studies and drafted the manuscript. FA designed the study and participated in the collection of clinical cases. KO contributed to the HPLC-fluorometric analyses and interpretation of the data. KK participated in the design of the study. FA and SaH supervised the experimental work, participated in the manuscript preparation and provided intellectual input in the study. SaH coordinated the group of investigators.

All authors have read and approved the final version of the manuscript.
